# Social Determinants of Health and Injury Among Children

**DOI:** 10.1001/jamanetworkopen.2025.13584

**Published:** 2025-06-04

**Authors:** Hunter Goodon, Justin P. Gawaziuk, Brenda Comaskey, Tracie O. Afifi, Dan Château, Marni Brownell, Jitender Sareen, Cora Morgan, Sarvesh Logsetty, Rae Spiwak

**Affiliations:** 1 Department of Surgery, Max Rady College of Medicine, University of Manitoba, Winnipeg, Manitoba, Canada; 2 Department of Community Health Sciences, Max Rady College of Medicine, University of Manitoba, Winnipeg, Manitoba, Canada; 3 Department of Psychiatry, Max Rady College of Medicine, University of Manitoba, Winnipeg, Manitoba, Canada; 4 Australian Institute of Health and Welfare, Canberra, Australia; 5 Manitoba Centre for Health Policy, Max Rady College of Medicine, University of Manitoba, Winnipeg, Manitoba, Canada; 6 Department of Psychology, University of Manitoba, Winnipeg, Manitoba, Canada; 7 Government of Manitoba, Winnipeg, Manitoba, Canada

## Abstract

**Importance:**

Pediatric physical injuries have lasting effects on child mental and physical health and social outcomes. Little is known about social determinants that increase the odds of injury in children.

**Objective:**

To examine the association between 14 social determinants of child health (SDoCH) and odds of pediatric injury.

**Design, Setting, and Participants:**

Population-based retrospective case-control study in Winnipeg, Canada. Cases (children aged ≤17 years admitted to the hospital from 2002 to 2019 with physical injuries) were linked to their mothers using a unique identifier and matched 1:5 on age, sex, and geographic region with uninjured controls from the general population. Data were analyzed from May 2023 to July 2024.

**Exposures:**

Fourteen SDoCH measured as present or absent from birth to date of injury: low-income neighborhood; rural status; receipt of income assistance; justice system involvement; parent with less than a high school education; social housing; having an immigrant parent; high residential mobility; being born to a teen mother; having a child in protective care; child mental health diagnosis; maternal axis I or axis II mental disorder; and maternal physical disorder.

**Main Outcomes and Measures:**

Pediatric injury that required hospitalization. Analyses were conducted using conditional multivariate logistic regression modeling.

**Results:**

The final groups included 9853 cases and 49 442 controls for a total sample of 59 295. For cases at time of injury, the mean (SD) age was 9.8 (5.2) years, 6358 (64.5%) were male, 4688 (47.6%) lived in a rural area, and 3639 (36.9%) were low income. There were no significant differences between cases and controls for demographics; however, there was a greater proportion than expected of both groups in the lowest quintile. In the final multivariable model, rural area (adjusted odds ratio [aOR], 6.62; 95% CI, 4.62-9.47), having a child in protective care (aOR, 1.43; 95% CI, 1.31-1.55), being born to a teen mother (aOR, 1.34; 95% CI, 1.26-1.41), parent criminal justice system involvement (aOR, 1.27; 95% CI, 1.21-1.33), and receipt of income assistance (aOR, 1.13; 95% CI, 1.06-1.21) increased odds of pediatric traumatic injury.

**Conclusions and Relevance:**

In this retrospective case-control study, several adverse SDoCH were associated with increased odds of pediatric injury. These findings can inform targeted injury risk reduction programs.

## Key Points

**Question:**

Using linked health and social administrative databases, which social determinants of child health (SDoCH) are associated with injuries among children?

**Findings:**

In this case-control study of 59 295 participants, rural area, protective care, being born to a teen mother, parent justice system involvement, and receipt of income assistance were associated with increased odds of pediatric injury; the other measured SDoCH were not.

**Meaning:**

These findings suggest that knowledge of SDoCH associated with increased odds of injury among children can be used to guide injury prevention efforts.

## Teaser

This case-control study evaluates the association between 14 social determinants of child health and odds of pediatric injury in Winnipeg, Canada.

## Introduction

Traumatic physical injuries are one of the leading causes of death in children in Canada and have significant societal and individual impacts. In 2018, over 20 000 children were hospitalized for injury in Canada and the total economic burden for preventable traumatic injury was $29.4 billion, including $20.4 billion in direct health care costs.^1^ Children living in low socioeconomic conditions^2,3,4^ and in rural areas^5,6^ are more likely to sustain an injury and the most common causes of injury in children are falls and transport injuries.^1^ Physical injuries in children can have lasting effects on their mental and physical health and social outcomes.^7,8^ With over 90% of unintentional childhood injuries being preventable, preinjury interventions informed by evidence are needed.^9^ Social determinants of child health (SDoCH) are a broad category of social and economic factors (eg, income, housing, parental health, and community resources) that influence a child’s health.^10^ While research has found associations between socioeconomic determinants (eg, poverty, low income, low education, and rural areas) and childhood injury,^2,3,4,5^ these studies lack examination of other factors influencing child health, such as residential mobility, protective care, immigrant parent, parent justice system involvement, and parental mental and physical health. These social determinants have been found to influence health status in childhood and across the lifespan.^11,12,13^ In addition, while recognized as measures of inequity and important influences on the life cycle of a child,^14,15^ these broader SDoCH have been infrequently used in the development of injury prevention programs.^16^

To address gaps in the literature and increase understanding of the influence of social factors on health outcomes, this retrospective case-control study used linked population-level data to identify which SDoCH increased the odds of physical traumatic injury requiring hospitalization among children in Manitoba, Canada. Our hypothesis was that odds of injury would be greater for children with adverse SDoCH compared with children without adverse SDoCH. Once identified, SDoCH that place children at increased odds of sustaining a physical traumatic injury were plotted in a Haddon matrix, a validated model used to identify sources and time periods of preventable injuries and potential points for interventions and prevention. This framework will address gaps in the literature and inform the development of targeted injury prevention programs for children.

## Methods

### Overview

The association between 14 SDoCH and pediatric traumatic injury was examined using social and clinical administrative datasets from the Manitoba Centre for Health Policy (MCHP) Population Data Repository. Children hospitalized for injury over a 17-year period (and matched controls) were linked to their mothers, and SDoCH were measured during the child’s lifespan from birth up to the date of injury. Reporting for this study follows the Reporting of studies Conducted using Observational Routinely-collected Data (RECORD) guidelines.^17^ Ethics approval for this study was obtained from the University of Manitoba Health Research Ethics Board (HREB). Informed consent from participants was waived by the HREB due to the use of fully anonymized administrative data.

### Data Sources

This study used linked population-level data from the Children’s Hospital of Winnipeg clinical trauma registry and the Population Health Research Data Repository located at the MCHP at the University of Manitoba. All children in the province of Manitoba with a significant injury are treated at the Children’s Hospital. The trauma registry includes date of injury, demographic factors (age and sex), injury-related factors (cause of the injury, such as motor vehicle), context of the injury (ie, recreational), and admission to an intensive care unit. The MCHP is a world-renowned data repository that receives deidentified provincial health service data supplemented by a variety of other social services and public use datasets, including vital statistics (birth and death records), census data, area-level income, immigration, education, family services, justice system, and the use of income assistance.^18^ A unique identifier, the personal health information number (PHIN), was used to link these health and social datasets (see eTable 1 in Supplement 1 for the list of datasets used).

### Study Groups

The injured cases included children (≤17 years of age) hospitalized with injuries over a 17-year period from 2002 to 2019. Cases were identified from the trauma registry and the date of injury was identified as the index date. Individuals hospitalized for self-harm injuries (*International Classification of Diseases, Ninth Revision, Clinical Modification [ICD-9-CM]* codes E950-E959 and *ICD-10-CM* codes X71-X83) were excluded as they differ in context from other causes of pediatric injury. To reduce selection bias and ensure the groups were comparable, cases were matched 1:5 with uninjured (before index date) controls from the general population based on sex, geographic region, and age at index date (49 442 participants). Geographic regions were based on 11 regional health authorities in the province of Manitoba (population ranging from <1000 in the Churchill Regional Health Authority to >750 000 for the city of Winnipeg).^19^ Study participants must have held a valid PHIN and Manitoba address for at least 1 year before the index date. Each patient PHIN was scrambled (sPHIN) to protect privacy, and deidentified data from the trauma registry was linked with the Population Health Research Data Repository. Children in both groups were linked to their mother using the sPHIN.

### Social Determinants of Child Health

Using linked clinical and repository data allows for the assessment of each child’s exposure (via linkage to their mother) to various SDoCH that may influence odds of injury in children. SDoCH used in this study (eTable 2 in Supplement 1) were low-income neighborhood, rural status, receipt of income assistance, parent justice system involvement, parent with less than a high school education, immigrant parent, high residential mobility, being born to a teen mother, child in protective care, child mental health disorder, maternal axis I or axis II mental disorder, and maternal physical disorder (see eTable 3 in Supplement 1 for mental and physical disorder *ICD* codes). SDoCH examined were based on previous work by our research team, MCHP examinations of social determinants of health, and a literature review.^20,21^ Children had variable exposure to SDoCH from their date of birth up to the date of injury. Individual SDoCH were binary variables and measured as present or absent (1) during the exposure period (parent high school completion, parent immigrant, and being born to a teen mother); (2) 3 or more times over the exposure period (high residential mobility); (3) 1 or more times over the exposure period (income assistance, social housing, child protective care, parent justice system involvement, child mental disorder, parent mental disorder (axis I or II), and parent physical disorder); or (4) measured at index date (residential postal code for rural or urban and income quintile).

### Statistical Analysis

Descriptive statistics for the 2 groups were calculated. A correlation matrix was generated to examine the overlap between SDoCH categories and guide model development. Conditional univariate analyses were performed on individual SDoCH measures using case or control as the binary response. To focus on identifying risk factors for injury, a conditional multivariable logistic regression model^22^ was created including SDoCH with an adjusted odds ratio (aOR) greater than 1 (95% CI) from the conditional univariate analysis. Degree of similarity between SDoCH variables guided appropriate variable selection for the statistical model. Correlation was defined as low (*r* = 0.10-0.29), medium (*r* = 0.30-0.49), high (*r* = 0.50-0.89), and very high (*r* ≥ 0.9).^23^ Statistical significance was defined at a more stringent *P* ≤ .01 due to the large sample size. Data analysis was performed using SAS version 9.4 (SAS Institute) from May 2023 to July 2024.

## Results

### Demographics

The final groups included 9853 cases and 49 442 controls for a total sample size of 59 295. For cases at time of injury, the mean (SD) age was 9.8 (5.2) years, 6358 (64.5%) were male, 4688 (47.6%) lived in a rural area, and 3639 (36.9%) were low income. Descriptive comparisons of injured children and their matches are shown in Table 1. There were no significant differences between cases and controls for age, sex, or geographic region, indicating successful matching. There were no differences in income level between the 2 groups; however, there was a greater proportion than expected of both groups in the lowest quintile (Q1). Table 1 also includes descriptive characteristics of injuries for the cases. The injury severity score (ISS) is an established score to assess trauma severity.^24^ In Canada, an ISS score greater than 12 is considered major trauma, and minor trauma is an ISS of 12 or less.

**Table 1.** Description of the Cohorts

Variable	Participants, No. (%)		χ^2^	*P* value
	Cases (n = 9853)	Controls (n = 49 442)		
Age, mean (SD)	9.84 (5.17)	9.83 (5.17)	−0.25^a^	.81
Sex				
Female	3495 (35.47)	17 554 (35.50)	0.004	.95
Male	6358 (64.53)	31 888 (64.50)		
Rural	4688 (47.60)	23 178 (46.88)	1.70	.19
Low income	3639 (36.90)	18 371 (37.16)	0.18	.67
Income quintile				
5 (Highest)	1759 (17.85)	9277 (18.76)	36.80	<.001
4	1727 (17.54)	8496 (17.18)		
3	1556 (15.79)	8562 (17.32)		
2	1858 (18.86)	9485 (19.18)		
1 (Lowest)	2800 (28.42)	13 024 (26.34)		
Income not found	153 (1.55)	598 (1.21)		
Injury characteristic				
Length of stay, mean (SD), d	3.3 (11.0)	NA	NA	NA
Injury severity score >12	5.4 (5.8)	NA	NA	NA
Injury type^b^				
Fall	4008 (40.68)	NA	NA	NA
Total transportation	1854 (18.81)	NA	NA	NA
Other land transport^c^	1080 (10.96)	NA	NA	NA
Bicycle	487 (4.94)	NA	NA	NA
Pedestrian	287 (2.91)	NA	NA	NA
Struck by or against	1242 (12.61)	NA	NA	NA
Suffocation and foreign body	772 (7.84)	NA	NA	NA
Assault	549 (5.57)	NA	NA	NA
Cuts and pierces	342 (3.47)	NA	NA	NA
Burns	309 (3.14)	NA	NA	NA
Overexertion	83 (0.84)	NA	NA	NA
Near drowning	55 (0.56)	NA	NA	NA
Unintentional firearm	47 (0.48)	NA	NA	NA
Other	592 (6.01)	NA	NA	NA

Abbreviation: NA, not applicable.

^a^ Calculated using *t* test.

^b^ Based on *International Statistical Classification of Diseases and Related Health Problems, Tenth Revision* codes.

^c^ Includes animals (riding or pulling), vehicles used in agriculture, and all-terrain or off-road vehicles.

### Correlation Matrix

A correlation matrix (eTable 4 in Supplement 1) was created to examine the overlap between SDoCH categories. Correlation was high between social housing and income assistance (*r* = 0.55) and medium between social housing and high residential mobility (*r* = 0.30), income assistance and residential mobility (r = 0.41), and income assistance and being born to a teen mother (*r* = 0.39). Due to the absence of very high correlation, the logistic regression model was created using all variables.

### Logistic Regression

Table 2 shows ORs for the conditional univariate logistic regression including all covariates. SDoCH associated with increased odds of childhood injury were rural area, protective care, moved frequently, social housing, income assistance, parent with less than a high school education, justice system involvement, being born to a teen mother, and maternal axis I mental disorder or physical disorder. A detailed description of preinjury maternal mental health can be found in eTable 5 in Supplement 1 and preinjury maternal physical health in eTable 6 in Supplement 1. Table 3 displays the multivariable conditional regression model including only covariates with an aOR greater than 1. In the final multivariable model, children who lived in a rural area (aOR, 6.62; 95% CI, 4.62-9.47), were in protective care (aOR, 1.43; 95% CI, 1.31-1.55), were born to a teen mother (aOR, 1.34; 95% CI, 1.26-1.41), had a parent involved in the criminal justice system (aOR, 1.27; 95% CI, 1.21-1.33), or received income assistance (aOR, 1.13; 95% CI, 1.06-1.21) were at increased odds of traumatic injury.

**Table 2.** Conditional Univariate Logistic Regression

Social determinant of child health	Estimate (SE)	Wald χ^2^	*R* ^2^	OR (95% Wald CI)	*P* value^a^
Rural status	1.734 (0.180)	92.96	0.0017	5.67 (3.98-8.06)	<.001
Child in care	0.584 (0.039)	225.89	<0.0001	1.79 (1.66-1.94)	<.001
Immigrant	−0.492 (0.064)	58.36	0.0011	0.61 (0.54-0.69)	<.001
Being born to a teen mother	0.439 (0.026)	286.66	0.0047	1.55 (1.48-1.63)	<.001
Income assistance	0.386 (0.027)	207.1	0.0034	1.47 (1.39-1.55)	<.001
Social housing	0.367 (0.033)	124.18	0.0020	1.44 (1.35-1.54)	<.001
Parent justice system	0.350 (0.024)	212.14	0.0035	1.42 (1.35-1.49)	<.001
Maternal axis II disorder	0.314 (0.241)	1.70	0.0000	1.37 (0.85-2.19)	.19
Parent <high school	0.246 (0.063)	15.06	0.0002	1.28 (1.13-1.45)	<.001
High residential mobility	0.230 (0.027)	72.69	0.0012	1.26 (1.19-1.33)	<.001
Child major mental disorder	0.182 (0.077)	5.53	0.0001	1.20 (1.03-1.4)	.02
Maternal axis I disorder	0.167 (0.024)	48.74	0.0008	1.18 (1.13-1.24)	<.001
Maternal physical disorder	0.089 (0.023)	15.54	0.0003	1.09 (1.05-1.14)	<.001
Low income	−0.027 (0.031)	0.751	0.0000	0.97 (0.92-1.03)	.39

Abbreviation: OR, odds ratio.

^a^ *P* < .01 indicates statistical significance.

**Table 3.** Conditional Multivariate Logistic Regression

Social determinant of child health	Estimate (SE)	Wald χ^2^	aOR (95% Wald CI)	*P* value^a^
Rural status	1.897 (0.183)	106.79	6.62 (4.63 to 9.48)	<.001
Child in care	0.355 (0.042)	71.99	1.43 (1.31 to 1.55)	<.001
Being born to a teen mother	0.291 (0.029)	103.65	1.34 (1.26 to 1.41)	<.001
Parental justice system	0.238 (0.025)	88.30	1.27 (1.21 to 1.33)	<.001
Income assistance	0.126 (0.034)	13.49	1.13 (1.06 to 1.21)	<.001
Social housing	0.082 (0.039)	4.36	1.09 (1.01 to 1.17)	.04
Maternal axis I mental disorder	0.064 (0.025)	6.66	1.07 (1.02 to 1.12)	.01
Maternal physical disorder	0.047 (0.023)	4.10	1.05 (1.00 to 1.10)	.04
Residential mobility	−0.036 (0.031)	0.51	0.98 (0.92 to 1.04)	.48
Parent <high school	0.015 (0.065)	0.06	1.02 (0.89 to 1.15)	.82

Abbreviation: aOR, adjusted odds ratio.

^a^ *P* < .01 indicates statistical significance.

### Injury Prevention: The Haddon Matrix

Using the results of this study to focus on children at greatest risk of injury would maximize the efficiency of prevention and intervention programs. The Haddon matrix is a validated model used to identify sources and time periods of preventable injuries and potential points for injury prevention. It is based on the premise that injuries result from harmful interactions between the individual, the agent, and the physical and socioeconomic environments.^25^ Application of a Haddon matrix divides the timeline into pre-event, event, and postevent interventions. Previous work by our team established a framework to organize strategies for pediatric burn injury interventions based on the Haddon matrix.^21^ We have expanded this preventative strategy to include a framework for all-cause pediatric injury based on findings in this study (Table 4 and Table 5). Table 5 presents unique interventions tailored to populations identified in this study with higher odds of injury; these can be used as stepping stones in the development of child injury prevention programs. In addition, upstream interventions at a policy level (eg, financial supports such as tax incentives for low-income families and increased investment in teen pregnancy prevention) would address some of the root causes of injury identified in this study.

**Table 4.** Haddon Matrix for Pediatric Injury

Time period	Human factors	Physical environment	Socioeconomic environment
Preinjury	1	2	3
Injury	4	5	6
Postinjury	7	8	9

**Table 5.** Interventions for Targeted Injury Prevention

Interventions	Targeted population	Haddon matrix	Active/Passive
Providing education about the importance of helmet use for bicycles and all-terrain vehicles.	Rural	1,2,4,5	Active
Provide easier access to and affordability of injury prevention devices through combined purchasing and installation subsidies (eg, smoke detectors, bicycle helmets, childproof locks, and life jackets).	Income assistance, child in care, and being born to a teen mother	2,3,5,6	Active and passive
Basic injury prevention and first aid education as part of “Families First” home visits for well babies.	Income assistance and being born to a teen mother	1,2,4,5,7	Active
Education for foster parents on injury prevention, first aid, and home safety.	Child in care	1,2,3,4,5,7	Active
Undertaking safety audits through home visits (eg, checking for safe storage of poisonous substances, properly functioning smoke alarms, gates on stairs).	Income assistance, child in care, being born to a teen mother, and parent justice system	1,2,3,4,5	Active and passive
Subsidized water and boat safety education and swimming lessons.	Rural, income assistance, child in care, and being born to a teen mother	1,2,3,4	Active
Education programming on safe farm equipment use, especially for boys.	Rural	1,2,4,5	Active
Injury prevention training for parents incarcerated or on parole.	Parent justice system	1,2,4,5	Active
Expanded childcare supports.	Being born to a teen mother	1,4,7	Active
Working with local Indigenous political leaders and organizations to design culturally appropriate injury prevention programs and first aid education.	Income assistance, child in care, and being born to a teen mother	1,2,4,5,7	Active
Basic injury prevention and first aid education as part of Aboriginal Head Start Program.	Income assistance, child in care, and being born to a teen mother	1,2,4,5,7	Active
Injury prevention at health care visits (eg, physicians and health care clinicians) including injury prevention education as part of routine checkups.	Rural, income assistance, child in care, and being born to a teen mother	1,2,3,4,5	Active

## Discussion

This study found that children who lived in a rural area, were in protective care, were born to a teen mother, had a parent involved in the criminal justice system, or who received income assistance had higher odds of being hospitalized for traumatic injury in Manitoba, Canada. Previous research found a relationship between children living in a rural area and increased odds of injury in Manitoba,^26^ Canada^5,6^ and the US.^5^ Our study confirms this finding with children living in a rural area having more than 6 times greater odds of injury. Factors that may contribute to these higher odds in rural areas include greater exposure to hazardous industries (eg, agriculture, mining, forestry, and fisheries), higher rates of off-road and/or all-terrain accidents and motor vehicle collisions (due to higher speeds, increased alcohol use, and lower use of restraints) and delayed trauma response.^5^ Children in protective care are known to have increased health concerns compared with children not in protective care. Beal et al^27^ found that 56.7% of youth aged 12 to 18 years in protective care engaged in high-risk behaviors with 39.6% involving substance use. Prior research has also indicated that children in protective care have higher rates of health care utilization and emergency department visits,^28,29^ with up to one-third of visits for injuries.^30^ We found children in protective care had nearly 1.5 greater odds of injury requiring hospitalization, suggesting a potential point of intervention for injury prevention programs (ie, educating foster care providers on injury prevention).

Children born to teen mothers have poorer health, worse educational and social outcomes, and lower cognitive development.^31^ In a Winnipeg study, Jutte et al^32^ found that children under 5 years born to teenage mothers had higher rates of hospitalization, and children of all ages had higher rates of death compared with children born to older mothers. In Sweden, Ekeus et al^33^ found that children of teenage mothers had a higher risk of hospital admission for violent injuries and unintentional injuries compared with mothers aged at least 32 years at the time of birth. The results of our study support these findings, demonstrating that children born to teen mothers have greater odds of physical traumatic injury. In Manitoba, teen birth rates are strongly associated with low income in both rural and urban areas.^34^ Teenage mothers likely experience more economic hardships than older mothers, leading to a stressful home environment, which can increase the risk for child injury. This finding suggests another point of intervention for targeted injury prevention programs (ie, educating teenage mothers on injury prevention at targeted periods, such as during well-child checkups). In contrast to the other SDoCH, this study found that children of immigrant parents had lower odds of injury compared with other children. This is consistent with other research^35^ and possibly due to a protective effect of less acculturation.^36^

The association between parental contact with the criminal justice system and odds of childhood injury is relatively unexplored. Arthur et al^37^ found that justice system involvement was an independent risk factor for emergency department visits for children under 17 years of age, and Whitten et al^38^ found an association between paternal and maternal criminal justice involvement and injury-related emergency department visits in children. Our study confirms this by finding that children with parental criminal justice involvement have an increased odds of physical traumatic injury. Contact with the justice system may signal a variety of familial, social, and economic hardships.^39^ Families who experience severe adversity may also have difficulties with parenting, reside in more hazardous environments, and have inadequate access to quality information on child health and injury prevention.^38,40^

Much evidence supports the association between low sociodemographic status and childhood injury; however, most studies examine socioeconomic status as a single indicator.^3,4,41^ There may be overlap between SDoCH, such as low-income neighborhoods, income assistance, and social housing. In our final model, children living in social housing and children from a low-income neighborhood were not found to have increased odds of injury, and children from families receiving income assistance had increased odds. This finding outlines the importance of isolating individual risk factors to determine the unique influence that each SDoCH has on child injury.

The final multivariate model did not find increased odds of injury for social housing, high residential mobility, parent with less than a high school education, maternal axis I mental disorder, or maternal physical disorder. It is possible the lack of association for these SDoCH may be due to how and when these were measured during the exposure period.

### Limitations and Strengths

This study has limitations. Use of administrative clinical data is limited to physician billing records based on treatment prevalence. In the final model, we failed to find an association between preinjury maternal mental or physical health and odds of pediatric injury; however, this may be due to mothers with young children being unable to seek treatment for mental or physical disorders due to managing busy households. In addition, physician billing in Manitoba only captured 1 *ICD-9 *or *ICD-10* diagnosis per outpatient visit at the time of the study, so some conditions may not be recorded. While hospital admissions do record multiple diagnoses, not all records may be entered in the patient medical record. This issue can lead to an underrepresentation of the proportion of children exposed to preinjury maternal mental and physical disorders. Some SDoCH we measured were fixed (eg, immigrant parent); however, others were measured only at the date of injury (eg, rural status) or across variable exposure periods, and it is unknown when a child may be most vulnerable to these determinants. Furthermore, if a parent SDoCH was measured outside Manitoba (eg, high school completion) or a parent left and returned over the course the exposure period, we would not have that information in the dataset. We did not examine injury type by SDoCH, and this could be a direction for future research. Due to the large number of analyses, there is a possibility of chance associations; however, we set a stringent *P* value for the large sample size to mitigate this.

We were unable to identify race or ethnicity, including Indigenous membership, due to lack of availability in the administrative data. Indigenous Canadians are overrepresented in many of the SDoCH categories that we did measure. In Manitoba, over 90% of children in care are Indigenous,^42^ and the teen pregnancy rate among First Nations in Manitoba is 6 times that of other Manitoba teens.^43^ Indigenous Canadians are overrepresented in the criminal justice system,^44^ and income gaps between Indigenous and other individuals persist.^45^ Therefore, injury prevention programs in Canada that address groups at higher risk of adverse outcomes need to consider the specific circumstances of Indigenous Canadians. In addition, structural determinants of health (eg, colonialism and discriminatory policies)^45^ need to be considered along with SDoCH for interventions to be successful at preventing childhood injury. This research has identified several SDoCH where structural barriers persist (eg, low income, justice system involvement, and disparities in how child protection agencies scrutinize and compensate families of origin vs foster care environments), and these may be stepping stones toward addressing structural determinants. We are developing partnerships with Indigenous groups to address these findings and enact change. Furthermore, these structural barriers are not unique to Manitoba; these results may be generalizable to other locales where Indigenous peoples are impacted by social and structural determinants of health.

A key strength of this study was the ability to link multiple administrative datasets from the MCHP with clinical data from the Children’s Hospital of Winnipeg trauma registry. The MCHP is world-renowned as a data source, composing over 99% of the population of Manitoba and unique in the breadth and depth of data available in a publicly-funded universal health care system.^46^ The Children’s Hospital of Winnipeg is a level I trauma referral center that captures most pediatric traumatic injury admissions in the province. We can examine hospitalizations, physician billing, and type of physician (field of specialist and primary care), which is essential for examining parental health as a SDoCH.^47,48^ Furthermore, the ability to link children to their mothers using the sPHIN enabled measurement of a wide range of SDoCH across numerous datasets. A final strength was the ability to match each of the injured children 1:5 with an uninjured child of the same age, sex, and geographic region, mitigating selection bias.

## Conclusions

Limited research examines the role of SDoCH and odds of injury in children. We conducted a 17-year retrospective case-control study matching injured children 1:5 with uninjured children from the general population and compared 14 measures of SDoCH between the 2 cohorts. Our study showed increased odds of injury for children in Manitoba, Canada, with the SDoCH of rural area, protective care, being born to a teen mother, income assistance, or parental criminal justice system involvement. Injury prevention programs should target these groups of children who have higher odds of adverse outcomes to maximize efficiency in both cost and prevention of injury.

## Notes

Supplement. **eTable 1.** Administrative datasets used in the study **eTable 2.** Social determinants of child health (SDoCH) used in the study **eTable 3. **International Classification of Diseases (ICD) codes **eTable 4.** Correlation matrix **eTable 5.** Univariate analysis of preinjury maternal mental health **eTable 6.** Univariate analysis of preinjury maternal physical health

Supplement 2.
Data Sharing Statement
