# Supplementary material for: Social Determinants of Health and Injury Among Children
Source: JAMA Netw Open. 2025 Jun 4;8(6):e2513584. doi: 10.1001/jamanetworkopen.2025.13584 (PMC12138722; doi:10.1001/jamanetworkopen.2025.13584)
Supplement: Supplement. — eTable 1. Administrative datasets used in the study eTable 2. Social determinants of child health (SDoCH) used in the study eTable 3. International Classification of Diseases (ICD) codes eTable 4. Correlation matrix eTable 5. Univariate analysis of preinjury maternal mental health eTable 6. Univariate analysis of preinjury maternal physical health [file jamanetwopen-e2513584-s001.pdf]

Supplemental Online Content

Goodon H, Gawaziuk JP, Comaskey B, et al. Social determinants of health and injury among children. *JAMA Netw Open*. 2025;8(6):e2513584.
doi:10.1001/jamanetworkopen.2025.13584

eTable 1. Administrative datasets used in the study

eTable 2. Social determinants of child health (SDoCH) used in the study

eTable 3. International Classification of Diseases (ICD) codes

eTable 4. Correlation matrix

eTable 5. Univariate analysis of preinjury maternal mental health

eTable 6. Univariate analysis of preinjury maternal physical health

This supplemental material has been provided by the authors to give readers additional information about their work.

© 2025 Goodon H et al. *JAMA Network Open*.

eTable 1. Administrative datasets used in the study

Database	Contents	Use for this proposed study
Pediatric Trauma Registry	Demographics, injury-specific details	Identification of individuals with trauma and the clinical factors that may influence outcomes of interest.
Manitoba Health Registry	Scrambled PHIN (sPHIN) for linkage, identify parents	Identification of pediatric trauma patients and their parents.
	Residential postal code	Identification of rural or remote residence.<br><br>Identification of high residential mobility.
Hospital Separations Abstracts	Admission and discharge date, diagnosis codes	Identification of hospitalizations for health disorders.
Medical Claims (physician billings)	Service dates and diagnosis codes	Identification of physician visits for health disorders.
Vital Statistics Mortality	Alive or deceased during study period	Identification of deceased status, date and cause of death.
Prosecutions Information Management System (PIMS)	Incident data (e.g., types and involvement)	Identification of individuals who have been involved in the justice system.
Employment / Income Assistance (SAMIN)	Receipt of income assistance	Identification of individuals who have received income assistance.
Child and Family Services: Applications and Intake	Child in protective care	Identification of children in care of child protective services.
Social Housing Tenant Management System (TMSI)	Residents of social housing units	Identification of individuals living in social housing units managed by Manitoba Housing.
Enrollment, Marks, and Assessments (STS/ICAB)	Education performance	Identification of individuals who have completed high school graduation.
Canada Census	Area-level income	Identification of lowest neighbourhood income quintile.
Immigration, Refugees and Citizenship Canada (IRCC) Permanent Resident Database	Immigration status	Identification of children with parent(s) not native to Canada.

© 2025 Goodon H et al. *JAMA Network Open*.

eTable 2. Social determinants of child health (SDoCH) used in this study

SDoCH	Description
Low-income neighbourhood	A child from a family belonging to the lowest-income quintile (derived from neighborhood census data).
Rural status	A child from a family residing in a region with a population of <10,000 people, based on residential postal code at index date.
Child from a family that has received income assistance	A child from a family that has received financial income assistance from the Employment and Income Assistance Program (EIA) in Manitoba.
Child with a parent involved in the justice system (as victim, witness or accused)	A child with a parent involved in the justice system based on the data acquired via Prosecution Information and Scheduling Management (PRISM) developed by Manitoba Justice Prosecution Service.
Child of a parent with less than high school completion.	Based on graduation status data from Manitoba Education.
Child with a parent who lived in social housing	A child from a family who has lived in social housing managed by Manitoba Housing and Community Development.
Child of a parent who immigrated	A child of parent(s) from a native country that is not Canada. Based on federal immigration data (IRCC) linked with MCHP data.
Child from a family with high residential mobility	A child of a family that has moved residences three or more times within 10 person-years, based on residential postal code.
Child of a teen mother	A child born to a mother who first gave birth at age 19 or younger.
Child mental disorder	A child with the presence of an Axis I mental disorder, based on ICD[a] diagnosis codes (anxiety, depression, substance use disorder).
Child of a mother with an Axis I mental disorder	A child of a mother with an Axis I disorder, based on ICD[a] diagnosis codes (anxiety, depression, substance use disorder).
Child of a mother with an Axis II mental disorder	A child of a mother with an Axis II mental disorder, based on ICD[a] diagnosis codes.
Child of a mother with a physical disorder	A child of a mother with a physical disorder diagnosis, measured using ICD[a] diagnosis codes (cardiovascular disease, cancer, chronic obstructive pulmonary disease, hypertension and diabetes).
Child in protective care	A child or sibling removed from family of origin and placed in the care of another adult due to concerns related to care, occurring at any time prior to index date. Based on data collected by Child and Family Services Information System (CFSIS).

[a]ICD = International Classification of Diseases Codes (see eTable 2 for full list of ICD codes used in the study).

© 2025 Goodon H et al. *JAMA Network Open*.

eTable 3. International Classification of Diseases (ICD) codes

Disorder	ICD Codes
**Axis I mental disorders[a]**	
Anxiety	ICD-9-CM: 300.0, 300.2, 300.3; ICD-10-CA: F40, F41.0, F41.1, F41.3, F41.8, F41.9, F42, F43.1
Depression	ICD-9-CM: 296.2, 296.3, 296.5,300.4, 309, 311; ICD-10-CA: F31.3-F31.5, F32, F33, F34.1, F38.0, F38.1, F43.2, F43.8, F53.0
Substance use disorders	ICD-9-CM: 291, 292, 304, 305, 303; ICD-10-CA: F10-F19, F55
**Axis II mental disorders[a]**	
Schizophrenic, episodic mood and delusional disorders; other nonorganic psychoses; pervasive developmental disorders	ICD-9-CM: 295 to 299.14
**Physical disorders[a]**	
Cardiovascular disease	ICD-9-CM: 410-414; ICD-10-CA: I20-I25
Cancer	ICD-9-CM: 140-208; ICD-10-CA: C00.0-C41.9, C45.0-C97
Chronic obstructive pulmonary disease (COPD)	ICD-9-CM: 491, 492, 494, 496; ICD-10-CA: J41, J42, J43, J44, J47
Hypertension	ICD-9-CM: 401-405; ICD-10-CA: I10-I13, I15
Diabetes	ICD-9-CM: 250; ICD-10-CA: E10-E14

[a]One or more hospitalizations and/or one or more outpatient visits (physician billing) pre-index date are considered a mental disorder or physical disorder diagnosis.

© 2025 Goodon H et al. *JAMA Network Open*.

eTable 4. Correlation matrix

	Rural	Low income	Child in care	Parent < high school	Social housing	Income assistance	Immigrant parent
**Rural**							
**Low income**	*r*=0.01<br>P= 0.006						
**Child in care**	*r*=-0.04<br>P<.0001	*r*=-0.1<br>P<.0001					
**Parent < high school**	*r*=-0.03<br>P<.0001	*r*=0.07<br>P<.0001	*r*=-0.07<br>P<.0001				
**Social housing**	*r*=-0.08<br>P<.0001	*r*=-0.16<br>P<.0001	*r*=0.26<br>P<.0001	*r*=-0.06<br>P<.0001			
**Income assistance**	*r*=-0.11<br>P<.0001	*r*=-0.23<br>P<.0001	*r*=0.33<br>P<0.0001	*r*=-0.11<br>P<0.0001	*r*=0.55<br>P<.0001		
**Immigrant parent**	*r*=-0.1<br>P<.0001	*r*=-0.04<br>P<.0001	*r*=-0.06<br>P<.0001	*r*=0.04<br>P<.0001	*r*=-0.05<br>P<.0001	*r*=-0.06<br>P<.0001	
**Parental justice system**	*r*=0.01<br>P=.0001	*r*=-0.11<br>P<.0001	*r*=0.18<br>P<.0001	*r*=-0.17<br>P<.0001	*r*=0.18<br>P<.0001	*r*=0.26<br>P<.0001	*r*=-0.09<br>P<.0001
**Child mental disorder**	*r*=-0.02<br>P<.0001	*r*=-0.001<br>P=0.8	*r*=0.06<br>P<.0001	*r*=0.01<br>P=0.001	*r*=0.03<br>P<.0001	*r*=0.04<br>P<.0001	*r*=-0.01<br>P=0.008
**Residential mobility**	*r*=-0.15<br>P<.0001	*r*=-0.12<br>P<.0001	*r*=0.28<br>P<.0001	*r*=-0.1<br>P<.0001	*r*=0.3<br>P<.0001	*r*=0.41<br>P<0.0001	*r*=-0.1<br>P<.0001
**Teen mother**	*r*=0.1<br>P<.0001	*r*=-0.23<br>P<.0001	*r*=0.28<br>P<.0001	*r*=-0.16<br>P<.0001	*r*=0.27<br>P<.0001	*r*=0.39<br>P<.0001	*r*=-0.08<br>P<.0001
**Maternal Axis I disorder**	*r*=-0.08<br>P<.0001	*r*=-0.02<br>P<.0001	*r*=0.15<br>P<.0001	*r*=-0.03<br>P<.0001	*r*=0.12<br>P<.0001	*r*=0.16<br>P<.0001	*r*=-0.04<br>P<.0001
**Maternal physical disorder**	*r*=-0.08<br>P<.0001	*r*=0.033<br>P<.0001	*r*=0.08<br>P<.0001	*r*=0.03<br>P<.0001	*r*=0.08<br>P<.0001	*r*=0.1<br>P<.0001	*r*=-0.02<br>P<.0001
**Maternal Axis II disorder**	*r*=0.00454<br>P=0.3	*r*=-0.01<br>P=0.005	*r*=0.05<br>P<.0001	*r*=-0.02<br>P<.0001	*r*=0.02<br>P<.0001	*r*=0.03<br>P<.0001	*r*=-0.007<br>P=0.08

© 2025 Goodon H et al. *JAMA Network Open*.

(continued)	Parent justice system	Child mental disorder	High residential mobility	Teen mother	Maternal Axis I disorder	Maternal physical disorder	Maternal Axis II disorder
**Rural**							
**Low income**							
**Child in care**							
**Parent < high school**							
**Social housing**							
**Income assistance**							
**Immigrant parent**							
**Parent justice system**							
**Child mental disorder**	*r*=0.01 P=0.002						
**High residential mobility**	*r*=0.2 P<.0001	*r*=0.01 P=0.01					
**Teen mother**	*r*=0.22 P<.0001	*r*=-0.005 P=0.20	*r*=0.25 P<.0001				
**Maternal Axis I disorder**	*r*=0.11 P<.0001	*r*=0.04 P<.0001	*r*=0.15 P<.0001	*r*=0.08 P<.0001			
**Maternal physical disorder**	*r*=0.05 P<.0001	*r*=0.03 P<.0001	*r*=0.07 P<.0001	*r*=0.02 P<.0001	*r*=0.16 P<.0001		
**Maternal Axis II disorder**	*r*=0.02 P<.0001	*r*=0.003 P= 0.46	*r*=0.02 P<.0001	*r*=0.03 P<.0001	*r*=0.06 P<.0001	*r*=0.02 P=0.0002	

© 2025 Goodon H et al. *JAMA Network Open*.

eTable 5. Univariate analysis of pre-injury maternal mental health

Mental Disorder	Maternal cases (N=9853) N (%)	Maternal controls (N=49,442) N (%)	Test statistic (χ^2)	Odds ratio (95% CI)	P value[a]
Anxiety	2209 (22.4)	9244 (20.1)	27.82	**1.15 (1.01-1.21)**	**<.0001**
Depression	1621 (16.5)	7155 (14.5)	25.55	**1.16 (1.09-1.23)**	**<.0001**
Substance abuse	514 (5.2)	1931 (3.9)	35.73	**1.35 (1.22-1.50)**	**<.0001**

[a]Statistical significance = p ≤.005

eTable 6. Univariate analysis of pre-injury maternal physical health

Physical disorder	Maternal cases (N=9853) N (%)	Maternal controls (N=49,442) N (%)	Test statistic (χ^2)	Odds ratio (95% CI)	P value[a]
Arthritis	2386 (24.2)	11,419 (23.1)	5.77	1.06 (1.01-1.11)	0.02
Cancer	164 (1.7)	874 (1.8)	0.51	0.94 (0.80-1.11)	0.48
Coronary heart disease	58 (0.6)	257 (0.5)	1.27	1.18 (0.89-1.57)	0.26
Diabetes	587 (6.0)	266 (5.3)	6.07	1.13 (1.03-1.24)	0.01
Hypertension	691 (7.0)	3720 (7.5)	3.11	0.93 (0.85-1.01)	0.08
Total respiratory morbidity[b]	1913 (19.4)	8429 (17.1)	31.97	**1.17 (1.11-1.24)**	**<.0001**

[a]Statistical significance = p ≤.005
[b]Total respiratory morbidity includes asthma, chronic or acute bronchitis, emphysema or chronic airway obstruction and chronic obstructive pulmonary disease.

© 2025 Goodon H et al. *JAMA Network Open*.
