# Supplementary material for: Social Determinants of Health and Injury Among Children
Source: JAMA Netw Open. 2025 Jun 4;8(6):e2513584. doi: 10.1001/jamanetworkopen.2025.13584 (PMC12138722; doi:10.1001/jamanetworkopen.2025.13584)
Supplement: Supplement 2. — Data Sharing Statement [file jamanetwopen-e2513584-s002.pdf]

Data Sharing Statement

Goodon. Social Determinants of Health and Injury Among Children. *JAMA Netw Open*. Published June 04, 2025. doi:10.1001/jamanetworkopen.2025.13584

Data
Data available: No

Additional Information
Explanation for why data not available: Data cannot be shared publicly due to privacy and access restrictions through the Manitoba Centre for Health Policy (https://umanitoba.ca/manitoba-centre-for-health-policy/data-repository) and the Provincial Health Research Privacy Committee (https://www.rithim.ca/phrpc-submission-requirements).
